# A Five-Year, *In Situ* Growth Study on Shallow-Water Populations of the Gorgonian Octocoral *Calcigorgia spiculifera* in the Gulf of Alaska

**DOI:** 10.1371/journal.pone.0169470

**Published:** 2017-01-09

**Authors:** Robert P. Stone, Patrick W. Malecha, Michele M. Masuda

**Affiliations:** Auke Bay Laboratories, Alaska Fisheries Science Center, National Marine Fisheries Service, National Oceanic and Atmospheric Administration, Juneau, Alaska, United States of America; Universita degli Studi di Genova, ITALY

## Abstract

Gorgonian octocorals are the most abundant corals in Alaska where they provide important structural habitat for managed species of demersal fish and invertebrates. Fifty-nine gorgonian species have been reported from Alaska waters but little is known about their life history characteristics to help us gauge their ability to recover from seafloor disturbance. Colonies of the holaxonian *Calcigorgia spiculifera* were tagged beginning in 1999 at three sites in Chatham Strait, Southeast Alaska, using scuba and their growth measured annually for up to 5 years. Colonies were video recorded, and computer image analysis tools provided calibration of video images for measuring the length of several branches. Growth data indicate that *C*. *spiculifera* grows much slower (6.0 mm yr^-1^) than other gorgonians in Alaska for which there are data and that intraspecific growth is highly variable. We fit a Bayesian linear mixed-effects model that showed that average colony growth was significantly reduced with warmer temperature and presence of necrosis. The model further indicated that growth may slow among larger (older) colonies. Based on these results and previous studies, we propose that gorgonian growth rates are taxonomically constrained at the Suborder level and that holaxonians grow the slowest followed by scleraxonians and calcaxonians (2–3 times as fast). Findings of this study indicate that it would take approximately 60 years for *C*. *spiculifera* to grow to its maximum size and depending on the location and size of the parental standing stock, at least one and possibly 10 additional years for recruitment to occur. Our results further indicate that colonies that are injured, perhaps chronically in areas of frequent disturbance, grow at slower rates and if the current trend of ocean warming continues then we can expect these corals to grow more slowly, and the habitats they form will require more time to recover from disturbance.

## Introduction

The recovery rate of disturbed benthic habitats in Alaska is of keen interest to fisheries managers since several studies have documented extensive disturbance to coral habitats from fishing activities [1–4]. While a dynamic model [5] to assess the effect of fishing gear on seafloor habitats has been developed and accepted by the North Pacific Fishery Management Council, application of the model is problematic given our limited knowledge of basic life history processes of emergent epifauna. Recovery rate is defined as the rate of change of an impacted habitat back to an un-impacted state following disturbance. Recovery rates of emergent epifauna such as cold-water corals depend on several factors, including individual growth rate, recruitment rate, and reproductive ecology. The ecosystem-level effects of disturbance to coral habitats are poorly understood but may be substantial given the reported slow growth rates, longevity [6–8], and reproductive traits of cold-water corals [9,10]. Injury, as evidenced by the presence of epibionts, reduces fecundity and lipid concentration in female *Paramuricea clavata* colonies in the Mediterranean as they allocate more resources into the recovery of tissue regeneration than reproduction [11].

At least 59 gorgonian species (Class Anthozoa, Subclass Octocorallia, Order Alcyonacea, Suborders Calcaxonia, Holaxonia, and Scleraxonia) inhabit Alaska waters, and approximately 63% [12] occur within the depth zone of current fishing activities (approximately 80–1000 m). Several species attain large size and/or form extensive coral garden or thicket habitats that provide essential habitat in the form of structure and refuge for some species of demersal fish and invertebrates [13, 1, 3, 4]. Most Alaska gorgonians inhabit depths which limit routine *in situ* observations and the collection of measurements; consequently, little is known about their life history. The gorgonian *Calcigorgia spiculifera* Broch, 1935, however, occurs at depths accessible with scuba, providing a unique opportunity to study the growth of this gorgonian *in situ*. *C*. *spiculifera* is one of 11 taxa in the Suborder Holaxonia and one of five taxa in the Family Acanthogorgiidae in Alaska waters. It is distributed from central British Columbia through the Gulf of Alaska (GOA) to the western Aleutian Islands at depths between 15 and 512 m [14, 15, 12]. It forms single-species assemblages in areas of bedrock outcropping in Southeast Alaska [7], the eastern GOA [4], and in the Aleutian Islands [1] where it is also a minor component of coral gardens [1, 3]. Our *in situ* observations indicate that *C*. *spiculifera* can reach a height and width of about 36 cm, and colonies become increasingly more arborescent and less uniplaner with increased size. Colonies have a “woody” axis, are quite flexible, and their carbonate skeletal components (including sclerites) are composed entirely of high-Mg calcite (R. Stone, unpublished data).

For this study we identified three shallow-water sites in inside waters of the GOA that support populations of *C*. *spiculifera*. Using scuba a total of 92 colonies were tagged and assessed annually for lateral branch growth over a 5-year period, of which 54 colonies provided adequate growth measurements for analysis. The goal of the study was to determine and model the growth rate of this gorgonian in shallow water and also to examine the effects of ocean temperature, colony health, and colony size on growth. Knowledge of the growth rate and the variables affecting growth of this gorgonian can lead to insights on its recovery from seafloor disturbance and also the effects of ocean warming on this species.

## Materials and Methods

### Study Sites

**Fig 1:**
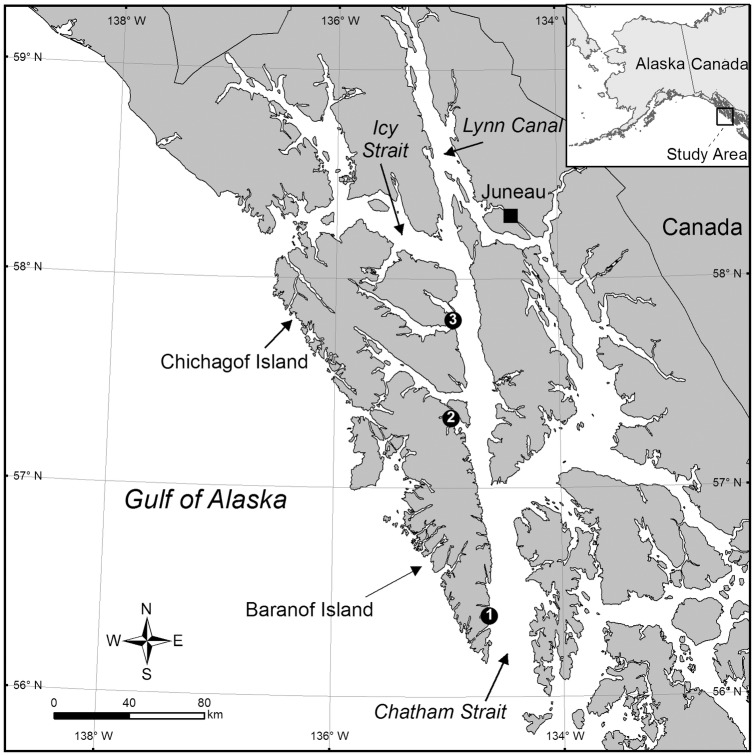
Map of Southeast Alaska showing the three sites where growth of the gorgonian coral *Calcigorgia spiculifera* was studied *in situ*.

The three study sites selected in the GOA were known from previous observations to support populations of *C*. *spiculifera* within scuba depth. All sites are located on the west side of Chatham Strait in Southeast Alaska (Fig 1). Chatham Strait is a very deep (~835 m), 240-km long passage that extends northward from the open ocean to the junction of Icy Strait and Lynn Canal (Fig 1). The strait, a major lateral strike-slip fault [16], is known as the Chatham Strait Fault [17]. The southern-most site, Site 1, is located at the base of a bedrock buttress northeast of Point Hutchinson (56° 23.299' N, 134° 38.152' W), Little Port Walter, Baranof Island (Fig 1). Site 2, farther north, is located near the entrance of the Middle Arm of Kelp Bay (57° 20.015' N, 134° 57.773' W), Baranof Island (Fig 1). This site, located on a massive bedrock outcrop approximately 100 m offshore, is characterized by the presence of more than 200 *C*. *spiculifera* colonies. Site 3, the northernmost site, is located just south of East Point (57° 48.218' N, 134° 56.750' W) at the north entrance of Tenakee Inlet, Chichagof Island (Fig 1). The three study sites are separated by approximately 172 km (111 km between Sites 1 and 2 and 61 km between Sites 2 and 3) and are subject to moderate tidal currents.

### Field Work

We used scuba to tag a total of 92 colonies at the 3 sites over the 5-year study, although only 54 colonies provided adequate growth measurements for analysis. All field work was conducted in State of Alaska waters and did not require any special permissions since no specimens were collected as a part of this research nor did the research involve endangered or protected species. Colonies were tagged above the holdfast with numbered plastic cinch-type tags. Healthy colonies with minimal sign of necrosis were selected for tagging. At Site 1, 26 colonies were initially tagged in July 1999, and 6 colonies were added to the study in July 2001. Depth of tagged colonies ranged 22–28 m (all depths reported in this manuscript have been adjusted to mean lower low water) and were attached to bedrock outcrop or large boulders (> 1 m diameter). At Site 2, 30 colonies were initially tagged in July 2001, and 5 additional colonies were tagged in early August 2003. Depth of tagged colonies ranged 19–22 m and were all attached to bedrock. At Site 3, 12 colonies were tagged in July 1999, and 3 additional colonies were tagged in July 2000. Depth of tagged colonies ranged 19–22 m; most colonies were attached to sloped bedrock although a few were attached to small boulders (< 1 m diameter). Consolidated substrate in the immediate area of all colonies was generally heavily encrusted with hydroids, bryozoans, and algae. Corals at all sites represented discrete patches that were restricted to an area of approximately 100–150 m^2^. All colonies were monitored for 2–5 years, until the end of the study in 2004.

**Fig 2:**
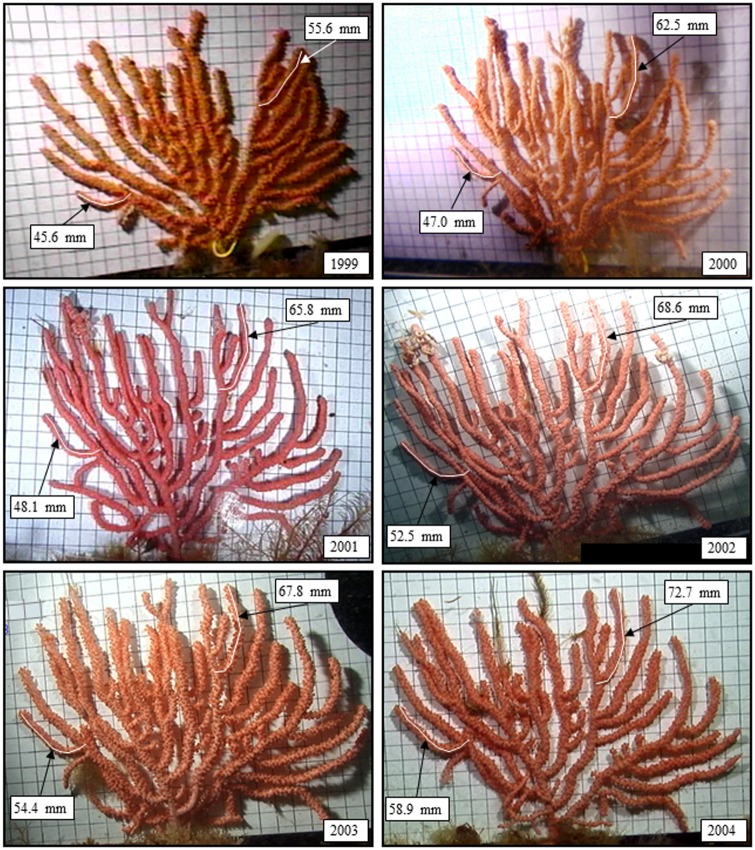
Series of photographs collected *in situ* of a coral colony at Site 1 showing the growth of two branches over a 5-year period (1999–2004). Colonies were video recorded while gently compressed between a sheet of clear acrylic glass and a rigid vinyl 1-cm grid.

Study sites were visited at approximately the same time each year. Divers surveyed the study area during each sampling event and initially placed weighted markers near the base of each tagged colony. Colonies were video recorded with either a Sony TR-700 Hi-8 (mention of trade names or commercial companies is for identification purposes only and does not imply endorsement by the National Marine Fisheries Service, NOAA) recorder (1999 only) or a Sony TRV-900 digital recorder set in progressive scan mode (all other years). Colonies were video recorded while gently compressed between a sheet of clear acrylic glass and a rigid, vinyl 1-cm grid (Fig 2). The compression aligned the branches evenly against the measuring plane. In the laboratory, computer image analysis tools [18] were used to capture and calibrate images of each colony and to digitally measure the length (excluding polyps) of several (n = 1–8) colony branches (Fig 2). Each branch was measured to the nearest millimeter along the medial axis from the point opposite its origin. Branches were chosen for measurement based exclusively on whether there were clear images of the branches in consecutive years. A branch length was computed as the average of three independent measurements of that branch made by the same person. Time between repeated branch length measurements was at least one day. We measured growth as the extension of branch tips (i.e. linear growth) between consecutive years rather than the more commonly provided measure of radial growth or the increase of the radius of the main axis. While the two measures are obviously related, radial growth is difficult to interpret in terms of recovery rate dynamics of these slow-growing animals unless extrapolated to the more relevant dimension (height or length) that provides the important structural habitat.

We defined annual growth as the change in branch length in one year; however, the time between consecutive sampling events was generally not exactly 365 days. For all sites, the number of days between sampling events averaged 364 days and ranged 319–416 days. To make annual growth measurements comparable, we standardized growth or the change in branch length to a fixed 365 days. We defined standardized length, *L*_*S*_, as

$$L_{S}=\frac{L_{T_{2}}-L_{T_{1}}}{T_{2}-T_{1}}\cdot 365\text{,}$$

where $L_{T_{i}}$ is the branch length measurement from sampling event *i*, and *T*_*1*_ and *T*_*2*_ are the Julian dates for consecutive sampling events, 1 and 2. The value $\frac{L_{T_{2}}-L_{T_{1}}}{T_{2}-T_{1}}$ is the average growth per day, and *L*_*S*_ is the growth per colony standardized to 365 days. These standardized growth measurements were used as the response variable in the modeling. Other measurements included the maximum height of each colony, generally along the central axis, made at the time of initial tagging. This measurement was used as the *height* variable in the modeling of growth. Also, the presence or absence of necrosis (0/1 variable in the model) was recorded for each colony each year.

Minilog 12-TR miniature microprocessor-controlled temperature loggers (VEMCO Ltd., 20 Angus Morton Drive, Bedford, Nova Scotia, Canada, B4B 0L9) were deployed at approximately 25 m depth at each study site. The loggers recorded temperature every 6 hours and have an accuracy of ±0.1°C. Four daily measurements were averaged to calculate a mean daily temperature from which an annual (365-day period) temperature was calculated based on the initial deployment date: Site 1 (8 July), Site 2 (15 July), and Site 3 (7 July). Salinity, temperature, depth and dissolved oxygen were measured at each study site during each sampling period with a Seabird Electronics Seacat Profiler.

Diver observations indicated that all three study sites had noticeably moderate to strong bottom currents implicating the importance of this oceanographic variable in community structure. Accordingly, we deployed a current meter at Site 1 to characterize the near-bottom currents typical of areas where these corals occupy. We measured water currents with a SonTek Argonaut-MD Doppler current meter between 10 April 2002 and 2 July 2002. The current meter, equipped with an internal compass, was moored 1 m off the bottom at a depth of 25 m. Three acoustic transducers generate short pulses of sound at known frequencies that propagate through the water and are reflected back to the meter, where the frequency change, or Doppler shift, is measured. The meter records current velocity (±0.5 cm s^-1^) and heading (±2°) of a 1-m cell of water 0.5 m above the seafloor. The current meter measures velocity and direction once per second. To preserve data memory space, the current meter was programmed to record average velocity and direction (based on 180 observations) every 15 minutes.

### Statistical Modeling

**Fig 3:**
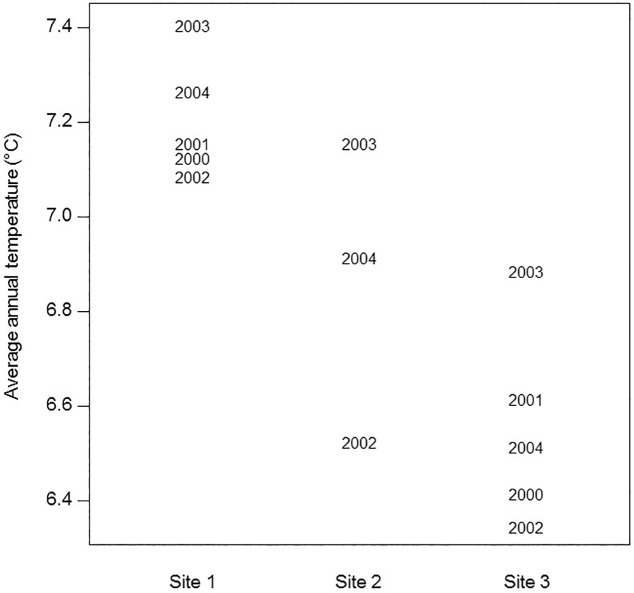
Average annual temperature (°C) measured at Sites 1–3. Points are indicated by the year the temperatures were measured.

We used a Bayesian linear mixed-effects model (BLMM) to model the average annual growth of colonies, also referred to as subjects, as a function of *temperature*, *necrosis*, and *height* and assumed normal sampling errors. With *temperature* in the model, we did not include the variable *study site* because of the variables’ relatedness. Average annual temperature differed by as much as one degree among sites (Fig 3), and since we were not interested in site differences per se, we included *temperature* and not *study site* in the model. The within-colony dependence of observations of the same colonies over time was modeled by including a colony-specific random intercept that represents the “baseline” average growth for each colony [19]. We defined the linear mixed-effects model (LMM) with a random intercept as the following:

$$y_{it}\;\sim \text{ Normal}\left(\mu_{it}\text{, }\sigma^{2}\right)\text{,}$$

$$\mu_{it}=\alpha_{i}+\beta_{1}\;\cdot {\text{ temperature}}_{it}+\beta_{2}\cdot {\text{necrosis}}_{it}+\beta_{3}\cdot {\text{height}}_{i}\text{,}$$

$$\alpha_{i}\;\sim \text{ Normal}\left(\theta \text{, }\sigma_{\alpha }^{2}\right)\text{,}$$

where *y*_*it*_ is the average growth of colony *i* in year *t*; *t* = 1, 2, …, *n*_*i*_; and *n*_*i*_ is the number of years that growth was measured for colony *i*. The precision of the sampling errors is defined as *τ* = (1/*σ*^2^). This model is a two-level hierarchical model where the observations are level 1 and the subjects or colonies are level 2. The vector of parameters, ***β***, shared by all subjects, describes how the subject response depends on the covariates given an underlying subject-specific response, *α*_*i*_, that persists over time. We assumed that the subject-specific random effect, *α*_*i*_, follows the normal distribution with unknown mean *θ* and variance $\sigma_{\alpha }^{2}$ and defined $\tau_{\alpha }=\left(1/\sigma_{\alpha }^{2}\right)$ to be the precision of the random effect. The model was fit with Bayesian methods, as implemented in software *WinBUGS* [20], and draws were simulated from the posterior distribution of unknown model parameters.

The regression parameters were given noninformative normal prior distributions with mean 0 and precision 0.0001 (inverse of the variance). We used a gamma distribution with small shape (0.001) and scale (0.001) parameters for precision *τ*. For the prior of the mean *θ*, we used a noninformative normal with mean 0 and precision 0.0001 and a uniform prior on the standard deviation *σ*_*α*_ on the interval [0, 100].

Using software *WinBUGS*, we ran three independent Markov chain Monte Carlo (MCMC) chains with different starting values. *WinBUGS* was run for 60,000 samples, and the first 30,000 samples of each chain were discarded as burn-in. Gelman and Rubin (G&R) shrink factors [21] were computed for each parameter to determine if the remaining 90,000 samples from the three chains showed evidence of convergence to the posterior distribution. All G&R values were < 1.2, which is consistent with convergence [22]. We made an additional check for convergence by calculating the ratio of the MCMC error and the sample standard deviation; a ratio of < 5% is consistent with convergence (all were < 5%; see *WinBUGS* User Manual).

Pooled MCMC samples after burn-in discard of model parameters were summarized with means, variances, and 95% probability intervals (PIs). Significance of a variable at the 0.05 level was determined from a 95% PI that did not include 0.

### Variance Partition Coefficient

Gelman and Pardoe [23] generalized the concept of *R*^2^ or the variation explained by the model in standard linear regression to multilevel hierarchical models with normal errors assumed at each level. Instead of fitting alternative models as is done in standard linear regression, the variation explained or *R*^2^ is computed at each level of the fitted hierarchical model, providing measures of relative importance of predictors and error at each level. We also calculated a pooling factor *λ* that Gelman and Pardoe [23] define as a measure of the degree of pooling at each level of the hierarchical model. We calculated *R*^2^ and *λ* from the Bayesian posterior simulation draws or MCMC samples, using *R* code based on the code provided in Appendix B of [23].

### Repeatability

#### Branch length

To assess the measurement error in repeated measurements of branch length, we applied the statistical tool of repeatability [24–26], also called intra-class correlation [27]. Branch length was digitally measured three, independent times by the same person from calibrated video images. Repeatability, a measure of the consistency of repeated measurements, provides an evaluation of the precision of measurements using our method and was calculated from three values for each branch. Specifically, repeatability is the proportion of the total variation accounted for by differences between individuals, or branches in our case, and is defined as

r=σA2(σA2+σW2),

where $\sigma_{A}^{2}$ is the between- or among-individual variation and $\sigma_{W}^{2}$ is the within-individual variation [24–27]. Repeatability values near 1 indicate highly consistent measurements for individuals. Conversely, repeatability values near 0 indicate the repeated measurements for an individual are no more similar than measurements from different individuals. To estimate the variance components, $\sigma_{A}^{2}$ and $\sigma_{W}^{2}$, used in calculating *r*, we fit an LMM for Gaussian data (Equations 8–10 in [26]), similar to the LMM fit to the growth data, using Bayesian methods implemented in software *WinBUGS*. The prior distributions for unknown parameters were set similarly to the ones used in the growth model. The model intercept was given a noninformative normal prior distribution with mean 0 and precision 0.0001. We used a gamma distribution with shape and scale parameters both set to 0.001 as the prior distribution for the within-individual precision $\left(\frac{1}{\sigma_{W}^{2}}\right)$ and a uniform prior on the interval [0, 100] for the between-individual standard deviation (*σ*_*A*_). Three independent MCMC chains were run for each year’s data for the sites combined, and the last 40,000 samples of each 160,000-sample chain were pooled to represent simulations from the posterior distribution. Convergence of the chains, after burn-in discard, to the posterior distribution was verified the same as described previously. We used the MCMC draws of $\frac{1}{\sigma_{W}^{2}}$ and, *σ*_*A*_ after burn-in discard, to calculate draws from the posterior distribution of *r* and used the mean as the estimate of *r*. Significance of *r* at the 0.05 level was determined from a 95% PI that did not include 0.

#### Branch growth

In this study, annual growth of a colony was determined from measurements on 1–8 colony branches. In addition to providing estimates of measurement error, repeatability can be used to estimate the consistency of phenotypes [26, 28]. Therefore, we also used repeatability to assess the variation in growth among branches of a colony. Repeatability values near 1 would indicate highly consistent growth among the branches of a colony, and conversely, values near 0 would indicate high variation in growth among branches of a colony. We estimated repeatability for branch growth in individual years with all sites combined. Colonies with only one branch measured in a year were excluded from the repeatability calculation (n = 0–3 colonies in any year).

## Results

### Oceanographic Conditions

Water temperatures differed between study sites, interannually, and generally increased during the study period (July 1999 to July 2004) with the lowest temperatures in 2002 and the highest in 2003 (Fig 3). Study sites had similar seasonal patterns of water temperature with the highest temperatures in late summer (August to mid-September) and a more variable timing of the annual minimum (January through early April). Seasonal temperature variability increased from the southernmost site (Site 1) to the northernmost site (Site 3) (Fig 3). Annual mean temperature ranged 7.09–7.41°C at Site 1, 6.53–7.16°C at Site 2, and 6.35–6.89°C at Site 3 (Table 1, Fig 3). Average daily temperature measurements at all sites ranged 3.63–11.08°C (Table 1) while measurements recorded every six hours at all sites ranged 3.50–11.90°C. Given the shallow-water nature of our study sites where temperatures are less stable and more extreme, these latter measurements likely represent the range of thermal tolerance for this species.

**Table 1:** Number of *Calcigorgia spiculifera* colonies (and total branches) for which growth measurements (mm) were made during each year at the three study sites. Growth per colony was calculated as the average growth of individual branches. SEM is standard error of the mean. Annual water temperature was recorded as the average of all daily temperature measurements at each site.

Site & year	No. of colonies (no. of branches)	Mean growth (SEM)	Growth range	Annual water temperature (daily range)
Site 1				
2000	9 (33)	6.35 (1.59)	0.23–14.99	7.13 (4.58–9.50)
2001	13 (46)	3.50 (0.78)	-0.71–9.50	7.16 (4.90–9.48)
2002	18 (69)	4.78 (1.08)	1.03–17.08	7.09 (4.40–10.23)
2003	17 (61)	2.04 (0.87)	-1.71–10.50	7.41 (4.60–10.33)
2004	18 (67)	3.92 (1.21)	-0.77–18.09	7.22 (4.40–10.80)
Site 2				
2002	13 (59)	9.25 (1.10)	3.37–13.33	6.53 (4.13–9.98)
2003	11 (53)	9.49 (1.61)	2.51–19.80	7.16 (5.15–10.05)
2004	15 (65)	8.22 (1.07)	0.03–13.62	6.92 (4.63–11.08)
Site 3				
2000	7 (21)	5.06 (2.03)	-1.76–13.86	6.42 (4.10–9.20)
2001	8 (23)	1.95 (0.67)	-0.41–5.76	6.62 (4.58–9.20)
2002	7 (22)	3.97 (1.82)	-0.29–14.18	6.35 (3.63–9.28)
2003	8 (24)	1.97 (0.86)	-0.20–4.50	6.89 (4.60–9.70)
2004	10 (35)	4.85 (1.10)	1.57–12.49	6.52 (3.78–10.80)

Overall, all sites were relatively saline and well oxygenated. During the sampling periods, salinity ranged from 31.21 to 31.82 PSU, and dissolved oxygen ranged from 5.55 to 7.76 ml/L (58–83% saturation) at Site 1. During the sampling periods, salinity ranged from 30.11 to 31.66 PSU, and dissolved oxygen ranged from 6.26 to 8.32 ml/L (65–89% saturation) at Site 2. At Site 3, salinity ranged from 30.61 to 31.59 PSU, and dissolved oxygen ranged from 6.10 to 6.87 ml/L (64–70% saturation) during the sampling periods.

Bottom currents measured at Site 1 for an 83-day period in April–July 2002 were relatively strong, tidally driven, and highly rhythmic. Current velocity ranged from 0.1 to 54.2 cm s^-1^ with an overall average velocity of 11.8 cm s^-1^. Current minima usually follow the predicted tidal extremes by a little over 4 hours. The weakest currents generally occur after low tides. Current maxima usually occur about 2 hours after the high tide and approximately concomitant with or shortly before the low tide. Current directions were mostly reciprocal to the southeast and northwest although the average current velocity was slightly higher for southeast currents (16.1 cm s^-1^ at 100°) than northeast currents (14.3 cm s^-1^ at 300°).

### Growth Measurements

The number of colonies providing quality video measurements varied from year to year, depending on colony behavior (e.g. feeding colonies with polyps extended hinder accurate measurement) and water visibility that affected the relocation of tagged colonies by divers. Depending on the year, we were able to collect growth measurements for 9 to 18 colonies at Site 1, 11 to 15 colonies at Site 2, and 7 to 10 colonies at Site 3 (Table 1). Of the 92 tagged colonies, 54 provided adequate growth measurements for analysis. The initial height of all colonies ranged from 89.6 to 289.6 mm, and 19 colonies (35%) developed necrosis during the study.

Growth of colonies varied between sites, interannually, and between colonies within a site. Mean growth (colonies pooled from all sites) was measured at 6.03 mm yr^-1^. Mean growth for Site 1 was 4.36 mm yr^-1^ and ranged from 2.04 (2003) to 6.35 mm yr^-1^ (2000). The total range of growth for all colonies in any year at Site 1 was -1.71 to 18.09 mm yr^-1^ (Table 1). Mean growth for Site 2 was the highest of all sites at 9.06 mm yr^-1^ and ranged from 8.22 (2004) to 9.49 mm yr^-1^ (2003). The total range of growth for all colonies in any year at Site 2 was 0.03 to 19.80 mm yr^-1^ (Table 1). Mean growth for Site 3 was the lowest of all sites at 4.08 mm yr^-1^ and ranged from 1.95 (2001) to 5.06 mm yr^-1^ (2000). The total range of growth for all colonies in any year at Site 3 was -1.76 to 14.18 mm yr^-1^ (Table 1).

### Growth Model

**Fig 4:**
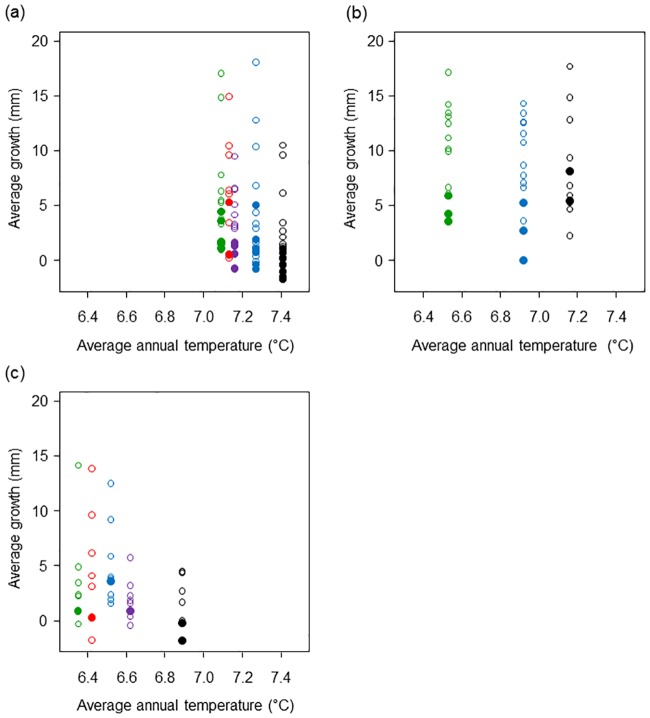
Average growth (mm) of colonies related to average annual temperature (°C) at (a) Site 1, (b) Site 2, and (c) Site 3. Average growth was repeatedly measured annually for the same colonies in 2000 (red), 2001 (purple), 2002 (green), 2003 (black), and 2004 (blue). Solid circles indicate colonies with necrosis.

The BLMM showed that average colony growth was significantly reduced with warmer temperature, presence of necrosis, and a larger initial height (Table 2). Variables *necrosis* and *temperature* were the most important related to average growth based on sizes of the estimated coefficients (Table 2). Magnitude of the estimated coefficients was similar for the two variables. According to the model, the presence of necrosis for a colony corresponds to an estimated decrease in average growth of 4.0 mm with all other variables fixed (Table 2). Colonies with observed necrosis in a given year are represented by solid circles and are generally at the lower end of average growth (Fig 4). According to the model, an increase of 1°C in average annual temperature corresponds to an estimated decrease in average growth of 3.6 mm with all other variables fixed (Table 2). The southernmost site, Site 1, was generally the warmest of the three sites during the study period, and colonies there showed the smallest average growth (Fig 4a). In general, average growth decreased with an increase in average annual temperature at all sites (Fig 4). Note that average growth repeatedly measured annually for the same colonies are represented by different colored circles in Fig 4. According to the model, variable *height* was significant (Table 2), and larger colonies at the onset of the study exhibited less growth. According to the model, an additional 1 mm of initial colony height corresponds to an estimated decrease in average growth of 3.1 × 10^−2^ mm with all other variables fixed (Table 2).

**Table 2:** Parameter estimates of the Bayesian linear mixed-effects model fit to the gorgonian growth data. Estimates were computed from 90,000 MCMC samples, using the software *WinBUGS*.

Parameter	Mean	Standard deviation	95% Probability Interval
*Temperature*	–3.6	1.2	(–5.9, –1.0)
*Necrosis*	–4.0	0.8	(–5.6, –2.3)
*Height*	–3.1 × 10^−2^	1.4 × 10^−2^	(–6.0 × 10^−2^, –3.0 × 10^−3^)
*σ*^2^	9.5	1.4	(7.1, 12.6)
*θ*	36.9	9.0	(17.1, 54.2)
_*$\sigma_{\alpha }^{2}$*_	11.2	3.5	(5.8, 19.2)

We calculated a fairly high *R*^2^ of 0.59 at level 1 of the hierarchical model, indicating that nearly 60% of the variation in the data was explained by the level-1 linear predictors common to all of the colonies: *temperature*, *necrosis*, and *height*. The pooling factor *λ* was estimated as 1.0, suggesting a high degree of population-level information in the data and a complete pooling of estimates toward a population mean. At level 2 of the model, *R*^2^ was calculated as 4.8 × 10^−7^ which indicates very little of the total variation was explained by the within-colony error which was not unreasonable given that colony growth was measured 5 years or less. The pooling factor *λ* at level 2 was also estimated as 1.0 since no additional pooling of estimates could be achieved.

Repeatability of branch length measurements was significant and consistently high every year: > 0.99, indicating very slight measurement error. The 95% PIs for *r* in individual years 1999–2004 were estimated as (0.9989, 0.9996), (0.9996, 0.9998), (0.9996, 0.9998), (0.9996, 0.9998), (0.9996, 0.9998), and (0.9995, 0.9997), respectively.

Repeatability for branch growth, or the proportion of variation accounted for by differences between colonies, was significant every year and estimated as 0.46, 0.41, 0.53, 0.61, and 0.63 for years 2000–2004, respectively. The 95% PIs for *r* in individual years 2000–2004 were broad: (0.13, 0.75), (0.15, 0.67), (0.36, 0.68), (0.45, 0.75), and (0.49, 0.76), respectively. Estimated repeatability values indicated “moderate repeatability” for branch growth according to an informal rating scheme put forth by Harper [25]. In 2000 and 2001, less than half of the total variation was accounted for by differences between colonies, indicating that more than half of the variation was contributed by natural within-colony growth variation among branches, in addition to very slight measurement error. In 2002–2004, more than half of the total variation was accounted for by between-colony variation, but variation in growth among branches of a colony was still sizable, supporting the idea that multiple branches of a colony should be measured for assessing a colony’s growth.

## Discussion

*Calcigorgia spiculifera* is the most common shallow-water (< 30 m depth) gorgonian in Alaska. We have observed patches in shallow water at many locations throughout Southeast Alaska, the Gulf of Alaska, and the central Aleutian Islands and in deeper water (to 512 m depth) throughout its range [12]. Populations in shallow-water areas of Southeast Alaska provided a rare opportunity to study the growth of this widely distributed coral on a broad geographical scale. The slow growth rate we measured (6.0 mm yr^-1^) corroborated results reported for the first year of the study (5.8 mm yr^-1^; [7]) and is noteworthy since it is the first for a holaxonian gorgonian and the slowest rate reported for any gorgonian in Alaska waters.

Growth rates have been determined for other Alaska species based on radiometric and skeletal growth pattern analyses. Growth rates for the calcaxonian gorgonian *Primnoa pacifica* were estimated at 17.4 mm yr^-1^ [6] and for the bamboo corals *Isidella tentaculum* and *Keratoisis* sp. at 14.0 mm yr^-1^ and 10.0 mm yr^-1^, respectively [8]. Using sclerochronology (ring counts), the growth rate of *Fanellia compressa* was estimated at 12.0–18.0 mm yr^-1^ (A. Andrews and R. Stone, unpublished data). Growth measurements have not been made for the few scleraxonian gorgonians known from the North Pacific Ocean; however, the growth rate for *Paragorgea arborea*, a large and important scleraxonian in Alaska has been estimated at 16.2 mm yr^-1^ using bomb ^14^C dating in the North Atlantic Ocean [29]. Furthermore, Sherwood and Edinger [29] measured growth rates for *Primnoa resedaeformis* (a calcaxonian) of 26.1 mm yr^-1^ and *Paramuricea* sp. (a holaxonian) of 5.6–5.8 mm yr^-1^. Based on the results of our work and previous studies, we propose that gorgonian growth rates are taxonomically constrained at the Suborder level and that holaxonians have the slowest growth rates followed by scleraxonians and calcaxonians (2–3 times as fast).

Several methods have been used to indirectly measure the growth rates of gorgonians, including sclerochronology, radiometric techniques, and radiocarbon (^14^C) dating (reviewed in [30]). The first two techniques are primarily limited to large gorgonians with heavily calcified axial skeletons and are difficult to perform on holaxonians that have hollow or semi-hollow axial skeletons. Bomb radiocarbon dating (^14^C) has been attempted for several species of calcaxonians in Alaska (*Primnoa pacifica* and *Fanellia compressa*) but was unsuccessful due to apparent complications with strong regional upwelling and depleted ^14^C sources at depth (A. Andrews and R. Stone, unpublished data). Previous research on gorgonian growth measured *in situ* has been accomplished by making direct measurements of branch or colony length with calipers or rulers [31–34]. Since these measurements were typically made to the nearest 5–10 mm the technique obviously would not have worked in this study. We are unaware of other studies which have used calibrated video images to digitally measure gorgonian growth *in situ*. This method has the advantage of providing a permanent record of colony morphometry and also greatly reduces diver time spent at depth. Precise measurements are possible with proper colony orientation with respect to the calibration grid and parallel alignment of the grid with the camera lens. One problem with our technique was that accurate measurements are difficult to obtain from colonies in which the polyps are extended while feeding. This was a problem for *C*. *spiculifera* which has relatively large polyps (approximately 1 mm in length) and multiple attempts (i.e. separate dives) were required during some sampling periods.

Repeatability, or the intraclass correlation, was an effective tool for assessing measurement error in repeated measurements of branch length. Our method of measuring growth from video images of colonies *in situ* was shown to produce highly consistent measurements in all years of the study (*r* > 0.99). This standard quantity for assessing measurement error has the added benefit that it can be used to compare the precision of measurements using our technique to the precision of other techniques. In addition to providing estimates of measurement error, repeatability can be used to estimate the consistency of phenotypes such as morphological, physiological, life-history, or behavioral traits [26, 28]. Therefore, we also used repeatability to assess the variation in branch growth within a colony. Although within-colony branch growth in our study was shown to be moderately repeatable, variation in growth among branches measured was still sizable, supporting the idea that growth is not uniform within a colony and any growth assessment should include multiple branches of a colony. The calculation of repeatability is straightforward, requiring estimates of variance components that can be obtained from various models and available software [26, 28].

Our findings of reduced growth for larger colonies and under warmer ocean conditions have previously been reported for other gorgonians. Growth rate (colony basal diameter) decreases with age in Mediterranean populations of red corals (*Corallium rubrum*; [35–37]). Branch extension rates showed no relationship to colony size in the common shallow-water Caribbean gorgonian *Plexaura flexuosa*, but whole colony relative growth decreased with increasing colony size presumably so that larger, more fecund colonies could allocate more energy for reproduction [38]. The Mediterranean gorgonian *Eunicella cavolini* also exhibits reduced growth with increasing colony size [39] and decreased growth during the warmer summer months [32]. The authors of that study speculated that decreased growth during the summer months was principally from high incident light intensities, however. The small negative growth rates measured for some branches in this study may have been an artifact of our measurement technique, although negative growth rates have previously been reported for other gorgonians [33]. Growth rate was variable for branches from the same colony and also between colonies (Table 1). Highly variable intraspecific growth rates have been reported for other gorgonians [33].

Shallow-water populations of *C*. *spiculifera* are not at risk to disturbance from commercial fisheries, but deeper water (> 80 m) populations are periodically disturbed in some regions [1, 3, 4]. Damage rates to this species from mixed but principally bottom trawl gear in the central Aleutian Islands were low at about 3% of the standing stock [1, 3], whereas no damage was observed to this species from long-line fisheries in the eastern Gulf of Alaska [4]. Corals in the Suborder Holaxonia (Families Acanthogorgiidae and Plexauridae) have axial skeletons that are at least partially hollow and without sclerites, providing a large degree of flexibility compared to corals in the Suborders Calcaxonia (Families Primnoidae, Isididae, and Chrysogorgiidae) and Scleraxonia (Family Paragorgiidae) that have solid, relatively inflexible axes without a hollow central core [40]. As pointed out previously [3] corals with flexible skeletons are less vulnerable to seafloor disturbance caused by fishing activities.

The degree to which gorgonians provide refuge to fish and their prey is largely dependent on their 3-dimensional structure and total surface area. Larger colonies simply provide more habitat; therefore, when assessing the recovery rate of gorgonian habitat from disturbance, the most appropriate parameter to measure is the time required to attain maximum size. The processes contributing to maximum size include both growth and recruitment; these processes are dependent on aspects of the reproductive ecology and how species respond to disturbance. The degree of disturbance may also greatly affect the rate of recovery. Areas of heavy disturbance where colonies are completely removed will require recruits from non-disturbed areas. In those areas, rates of recovery will depend initially on the rate of recruitment and then on growth to maximum size. In areas where colonies are damaged but otherwise intact, recovery processes will depend on the ability of colonies to reproduce while regenerating tissue. We observed very few colonies smaller than 100 mm at the study sites during the entire study period, indicating that recruitment for this species is quite limited, at least at our three study sites, and is a rare and sporadic event not occurring annually but more likely on the order of decades. Samples from large specimens (> 20 cm height) collected at Site 2 in August 2003 (as part of a separate, independent study) indicate that this species is a gonochoristic (i.e. separate genders) brooder that appears to breed at least annually. The presence of large, heavily yolk-laden embryos and planula larvae in the female polyps indicates a late summer or early fall recruitment. So the factors that are limiting recruitment at our study sites do not appear to be inadequate spawning biomass or the lack of adequate recruitment habitat but some other factor, possibly early settlement predation [41].

The most relevant question regarding recovery of a particular habitat is how much time is required for the dominant, usually the largest or most abundant, structure-forming species in a community to recover to its pre-disturbance state. The findings of this study indicate that approximately 60 years would be required for *C*. *spiculifera* to grow to its maximum size and depending on the location and size of the parental standing stock at least one and possibly 10 additional years for recruitment to occur. A 20-cm high colony, presumably the size-at-first reproduction based on our limited sampling and the size at which this species may provide “functional habitat”, that is structural habitat equivalent to maximum size, would require 33 years (plus at least one year for recruitment) to become established after removal from the seafloor. Our results further indicate that colonies that are injured, perhaps chronically in areas of frequent disturbance, grow at slower rates. If the current trend of ocean warming continues, then we can expect these corals to grow more slowly and habitats dominated by corals will require even longer periods of time to recover from disturbance.
